# Endophytic *Metarhizium robertsii* suppresses the phytopathogen, *Cochliobolus heterostrophus* and modulates  maize defenses

**DOI:** 10.1371/journal.pone.0272944

**Published:** 2022-09-22

**Authors:** Imtiaz Ahmad, María del Mar Jiménez-Gasco, Dawn S. Luthe, Mary E. Barbercheck

**Affiliations:** 1 Department of Entomology, The Pennsylvania State University, University Park, Pennsylvania, United States of America; 2 Department of Plant Pathology and Environmental Microbiology, The Pennsylvania State University, University Park, Pennsylvania, United States of America; 3 Department of Plant Science, The Pennsylvania State University, University Park, Pennsylvania, United States of America; Government College University Faisalabad, PAKISTAN

## Abstract

Fungi in the genus *Metarhizium* (Hypocreales: Clavicipitaceae) are insect-pathogens and endophytes that can benefit their host plant through growth promotion and protection against stresses. *Cochliobolus heterostrophus* (Drechsler) Drechsler (Pleosporales: Pleosporaceae) is an economically-significant phytopathogenic fungus that causes Southern Corn Leaf Blight (SCLB) in maize. We conducted greenhouse and lab-based experiments to determine the effects of endophytic *M*. *robertsii* J.F. Bisch., Rehner & Humber on growth and defense in maize (*Zea mays* L.) infected with *C*. *heterostrophus*. We inoculated maize seeds with spores of *M*. *robertsii* and, at the 3 to 4-leaf stage, the youngest true leaf of *M*. *robertsii*-treated and untreated control plants with spores of *C*. *heterostrophus*. After 96 h, we measured maize height, above-ground biomass, endophytic colonization by *M*. *robertsii*, severity of SCLB, and expression of plant defense genes and phytohormone content. We recovered *M*. *robertsii* from 74% of plants grown from treated seed. The severity of SCLB in *M*. *robertsii*-treated maize plants was lower than in plants inoculated only with *C*. *heterostrophus*. *M*. *robertsii-*treated maize inoculated or not inoculated with *C*. *heterostrophus* showed greater height and above-ground biomass compared with untreated control plants. Height and above-ground biomass of maize co-inoculated with *M*. *robertsii* and *C*. *heterostrophus* were not different from *M*. *robertsii*-treated maize. *M*. *robertsii* modulated the expression of defense genes and the phytohormone content in maize inoculated with *C*. *heterostrophus* compared with plants not inoculated with *C*. *heterostrophus* and control plants. These results suggest that endophytic *M*. *robertsii* can promote maize growth and reduce development of SCLB, possibly by induced systemic resistance mediated by modulation of phytohormones and expression of defense and growth-related genes in maize.

## Introduction

Plants face various biotic and abiotic challenges such as diseases, insect pests, and water and nutrient deficiencies that can significantly decrease crop yield and quality [1]. To overcome such challenges, growers typically apply synthetic pesticides and fertilizers. While these materials can protect crops from stress, their extensive use has been associated with risks to environmental sustainability and human health [2]. To defend against biotic and abiotic stresses, multilayered defense strategies have evolved in plants [3–5], including mutualistic associations with microbes [6–9]. Soilborne microbes interact with plants, improving their fitness [10–12] and are key drivers and modulators of plant diversity and productivity [13, 14]. Endophytic insect-pathogenic fungi (EIPF) occur naturally in soil as rhizospheric and/or endophytic microbes in managed and unmanaged habitats [15]. They can infect insects and plants directly, and when associated with plants, provide multiple benefits including plant growth promotion and nutrient transfer [16–18], plant disease suppression [19–22], and insect growth suppression [4, 23, 24].

EIPF in the genus *Metarhizium* (Hypocreales: Clavicipitaceae) have a broad arthropod host range and are well-adapted to agroecosystems [25–29]. Several *Metarhizium* spp. naturally associate with the roots of grasses, shrubs, herbs, and trees under field conditions [30, 31]. Experimentally, multiple species of *Metarhizium* colonize the roots of many plant species, including switchgrass, haricot bean, wheat, and soybean and promote plant growth [4, 7, 16, 32–36]. For example, plant growth promotion has been observed for multiple species of *Metarhizium* spp. in tomato [34], maize [4, 7, 37, 38], soybean [39], peanut [40], potato [41], cassava [35], sweet pepper [42], switchgrass and haricot beans [36].

Mutualistic plant-microbe interactions can induce phytohormone defense mechanisms against herbivores in plants [43, 44]. In general, the jasmonic acid (JA) and salicylic acid (SA) pathways modulate plant defense [45–50]. Beneficial plant-microbe interactions, including those involving fungi, can modulate SA and JA pathways [4, 51–53]. For instance, Rivas-Franco et al. (2020) reported increased levels of SA and JA in maize roots endophytically colonized by *M*. *anisopliae* [54–57]. Plant-fungal symbioses may result in modulation of the defense signaling cascade as an alternative adaptive strategy to cope with hostile environments. Such responses by plants involve sensitizing defense reactions to harsh conditions in the absence of a challenge (trigger of stimulus). This process is referred to as ‘priming’ [58–60]. Other phytohormones can be affected by endophytic and pathogenic colonization of plants. For example, abscisic acid (ABA) is a vital phytohormone induced in response to various biotic and abiotic stresses [61]. Under salinity stress, *Metarhizium*-inoculated soybeans showed higher JA levels and lower ABA compared to the non-inoculated control suggesting mitigation of salinity stress in *M*. *anisopliae* inoculated-plants [62]. Gibberellins (GA) are primarily involved in plant growth regulation [63] but recent reports revealed that they also regulate certain biological processes in response to stress [64]. Gibberellins and ABA antagonistically mediate many plant developmental processes [65].

Southern Corn Leaf Blight (SCLB), caused by the phytopathogen *Cochliobolus heterostrophus*, is regarded as one of the most destructive foliar diseases of maize due to its extensive impact on crop yield and quality [66]. Biological control, including the use of soil microorganisms to control plant diseases, offers an attractive alternative to management of plant disease with pesticides. There is increasing interest in understanding the role of and potential for exploiting the soil microbial community, and of fungal endophytes, specifically, to enhance plant productivity and tolerance to insect pests and phytopathogens in agricultural systems [67, 68]. The ability to predictably exploit soil microorganisms for biological control will require a better understanding of defense modulation of host plants induced by endophytic fungi generally, and *Metarhizium* spp. particularly.

Here we determined the effects of endophytic *M*. *robertsii* on indicators of maize growth and defense and on the severity of disease caused by *C*. *heterostrophus*. Our specific objectives were to assess the effect of endophytic *M*. *robertsii* on: 1) the severity of SCLB caused by *C*. *heterostrophus*; 2) the modulation of phytohormone content in maize with and without SCLB; and 3) the expression of key defense genes in maize with and without SCLB. We hypothesized that endophytic *M*. *robertsii* will suppress the severity of SCLB caused by *C*. *heterostrophus*. We also hypothesized that endophytic *M*. *robertsii* will modulate the expression of defense genes and phytohormone content in maize in response to the infection by *C*. *heterostrophus*.

## Materials and methods

### Fungal isolates

We used an isolate of *M*. *robertsii* J. F. Bischoff, Rehner & Humber originally collected from a field experiment designed to determine the benefits and trade-offs of cover crop diversity on a suite of ecosystem functions in an organic agronomic grain production system [27]. We obtained the isolate by sentinel insect baiting of soil with *Galleria mellonella* [69] and obtained pure cultures by culturing single conidia from sporulating *G*. *mellonella* cadavers on dodine-free semi-selective CTC medium [70].

We confirmed the identity of *M*. *robertsii* using routine morphological and molecular methods [71, 72]. We stored conidia of single spore isolates of *M*. *robertsii* on beads (Pro-Lab Diagnostics Microbank™ Bacterial and Fungal Preservation System) at -80°C for use in experiments. We submitted the translation elongation factor 1-alpha (TEF1-alpha) sequence of *M*. *robertsii* to NCBI GenBank under accession number MK988559 and the single spore isolate culture to The Agricultural Research Service Collection of Entomopathogenic Fungal Cultures (ARSEF) under the accession number 14325.

To produce inoculum of *M*. *robertsii*, we transferred beads from cryovials onto PDA medium and incubated the plates at 25 ± 2°C in the dark for ~14 days. We harvested the conidia under aseptic conditions and suspended them in a sterile 0.1% aqueous solution (v/v) of Triton™ X-100 (Dow Chemical Co., Midland, MI). We homogenized the conidial suspension by vigorously shaking for one minute and filtered the homogenized conidial suspension through four layers of sterile cheese cloth to separate the fungal mycelium fragments from conidia and determined the concentration of the stock conidial suspension under a compound microscope at 400X magnification with a Neubauer hemocytometer. We adjusted the concentration of *M*. *robertsii* to 1 x 10^8^ conidia ml^-1^ for seed inoculation. To determine the viability of the conidia, we assessed their ability to form a germ tube by plating 80 μl of the conidial suspension onto PDA medium and incubating in the dark at 25 ± 2°C for 24 h, then calculated percent viability by randomly counting 200 conidia at 400X magnification. We considered conidia viable if hyphae were visible or the germ tube was at least twice the length of the conidium. We only used conidial suspensions with germination rates of greater than 90% in experiments.

We obtained *C*. *heterostrophus* from Dr. Surinder Chopra in the Department of Plant Science at Penn State University, USA. We produced inoculum and evaluated the viability of conidia of *C*. *heterostrophus* by the method described above for *M*. *robertsii*. We adjusted the concentration of *C*. *heterostrophus* to 1 x 10^5^ conidia ml^-1^ for leaf inoculation with a 250 ml spray bottle.

### Greenhouse experiment

#### Surface disinfection of maize seeds

We surface disinfected seeds (*Zea mays* var. ‘Blue River LT671669’, organic) in a sterile laminar flow hood by immersion in 0.1% sodium hypochlorite for two minutes followed by soaking in 70% ethanol for three minutes and rinsing three times in sterile distilled water [68]. To confirm successful surface disinfection, we placed three randomly selected seeds onto a Petri plate (100 x 15 mm) containing Sabouraud dextrose agar (SDA) medium. We plated 50 ml of the final rinse water onto Petri plates containing SDA and incubated them in darkness at 25 ± 2°C for ~10 days. Surface disinfection was considered successful if no microbial growth was observed on the plates after 10 days.

#### Soil preparation

We prepared plant growth medium by mixing steamed field soil and potting mix (Vigoro Industries, Inc., Northbrook, IL) in a 1:1 ratio (v/v). We steamed the growth medium twice for 2 h at ~120°C in a steam sterilizer to reduce the prevalence of other microbes in the plant growth medium. After steaming the medium, we waited ~48 h before using it in experiments to avoid toxicity to plants.

#### Seed treatment

To inoculate the surface-disinfected seed with *M*. *robertsii*, we placed seeds in 100 ml of freshly prepared conidial suspension (1 x 10^8^ conidia ml^-1^) in a 250 ml sterile beaker and non-inoculated control seeds in a 250 ml beaker containing 100 ml of 0.1% Triton X-100 aqueous solution and covered them with aluminum foil. We placed the beakers containing the inoculated and non-inoculated seeds on a shaker at 10 rpm for 2 h and then planted the seeds directly from beakers with sterile spatulas.

#### Plant pot preparation

We filled steam-sterilized plastic pots (15 cm diam x 14.7 cm deep) with the prepared growth medium. Into each prepared pot, we planted one *M*. *robertsii*-treated or non-treated maize seed at a depth of ~2.5 cm. We prepared ~30 to 35 pots for each of four treatments and repeated the experiment twice. The four treatments included: 1) plants grown from *M*. *robertsii*-treated seed; 2) plants grown from *M*. *robertsii-*treated seed and inoculated with *C*. *heterostrophus;* 3) plants grown from grown untreated seed and inoculated with *C*. *heterostrophus;* and 4) an untreated control. Each treatment was represented by a total of 30 to 35 maize plants.

#### Inoculation of plants with *C*. *heterostrophus*

We placed the prepared pots randomly on a greenhouse bench with 16L:8D photoperiod at 25 ± 3°C and provided water equally as needed, approximately 2–3 times per week. At maize growth stage ~V3-V4 (~21 days after germination), we inoculated plants randomly assigned to the *C*. *heterostrophus-*only and the *C*. *heterostrophus + M*. *robertsii* treatments with *C*. *heterostrophus* and plants randomly assigned to the *M*. *robertsii-*only or untreated control with an aqueous solution of 0.1% Triton X-100 with a 250 ml sprayer bottle to run-off. We covered all plants with clear plastic sheeting for 96 h to maintain humidity to facilitate *C*. *heterostrophus* infection.

#### Plant response

We terminated the experiment ~96 h after inoculation with *C*. *heterostrophus* when maize plants were at growth stage V4 to V5. For all plants, we measured height from the base of the plant to the tip of the longest fully emerged true leaf and above-ground biomass by cutting the plant at the soil surface with clean scissors. We collected two, 5-cm long root sections from each plant to assay for endophytic colonization by *M*. *robertsii*. The fourth true leaf was removed from each plant for analysis of disease severity, endophytic colonization by *M*. *robertsii*, defense gene expression and phytohormone content. Approximately ~100–150 mg of the fourth true leaf was removed and placed into pre-labeled 2 ml Eppendorf tube, flash frozen in liquid nitrogen, and then stored at -80°C until processing for gene expression and phytohormone content. To determine biomass of each plant, we placed the remaining plant tissue in separate dried and pre-weighed brown paper bags, and oven-dried the plant material at 60°C for ~21 days, when the dried plant material was weighed.

#### Estimation of disease severity

We measured the severity of SCLB by scanning the fourth true leaf of each plant and measuring the percentage of leaf area covered by lesions caused by SCLB using ImageJ version 1.53 [73].

#### Endophytic colonization by *M*. *robertsii*

We evaluated the endophytic colonization of leaf and root tissue from each maize plant. The two 5-cm long primary root sections excised from each plant were rinsed with tap water to remove soil. We surface disinfested the excised leaf and root sections individually by submerging in 0.5% sodium hypochlorite for three minutes followed by 70% ethanol for three minutes, followed by serially rinsing three times in sterile deionized water. To confirm tissue disinfestation, we plated 50 μl of the final rinse water onto SDA medium and incubated the dishes at ~25 ± 2°C for 10 days in darkness. We cut off ~1 mm of outer edges of the surface disinfested leaf and ends of the root tissues using sterile dissecting scissors to remove dead cells and cut each leaf section into six, 6 x 6 mm sections and each root section into three, 6 mm long sections so that each plant generated six leaf and six root sections. We plated each tissue type from each plant in a labeled petri dish prepared with CTC medium by pressing the tissue flat against the surface of the medium. The plates were sealed with parafilm and incubated in dark at 25 ± 2°C for 14 days. We identified *M*. *robertsii* by characteristic white hyphal growth and dark green conidia and then cultured fungi emerging from the plant sections to confirm their identity as *M*. *robertsii* by molecular methods [72]. We considered a plant to be endophytically colonized when we observed growth of *M*. *robertsii* from one or more root or leaf sections. We calculated the proportion of endophytic plants by dividing the total number of colonized plants by the total number of *M*. *robertsii*-treated plants.

#### Phytohormone profiles and defense-related gene expression in maize

To analyze maize defense gene expression, we homogenized ~100 mg of the leaf tissue in liquid nitrogen (GenoGrinder 2000, OPS Diagnostics). We extracted RNA with 1 mL of TRIzol (Life Technologies, USA) per ~100 mg of tissue. The genomic DNA-free RNA was quantified (Nanodrop, Thermo-Fisher Scientific), and 1 μg of total RNA was used to prepare complementary DNA (cDNA) by using High Capacity cDNA Reverse Transcription kit (Applied Biosystems). Then, qRT-PCR was performed (7500 Fast Real-Time qPCR, Applied Biosystems, ThermoFisher Scientific, Inc.) with Fast Start Universal SYBR Green Master Mix (Roche Molecular Systems, Inc.) with actin as a reference gene and gene-specific primers (S1 Table).

The phytohormone profiling of pre-weighed maize leaf tissue was performed by the Proteomic and Metabolomic Facility of The Nebraska Center for Biotechnology at The University of Nebraska, Lincoln.

### Statistical analyses

We performed all statistical analyses in JMP^®^ Pro 16.0.0 (SAS Institute Inc., Cary, NC). We used mixed model ANOVA for all response variables and designated all treatment variables as fixed factors and block (trial replicate number) as a random factor. When the model was significant, we used Tukey’s honest significant difference *post-hoc* test of means. We considered results of analyses significant at P < 0.05. For all analyses, we transformed proportions using the square root arcsine to meet assumptions of normality, equality of variances and to reduce heterogeneity of variances [74]. Data presented in figures and tables are not transformed.

## Results

### Endophytic colonization and maize growth

We recovered *M*. *robertsii* from 74% of ~V4-V5 maize plants grown from *M*. *robertsii*-treated seed.

**Fig 1:**
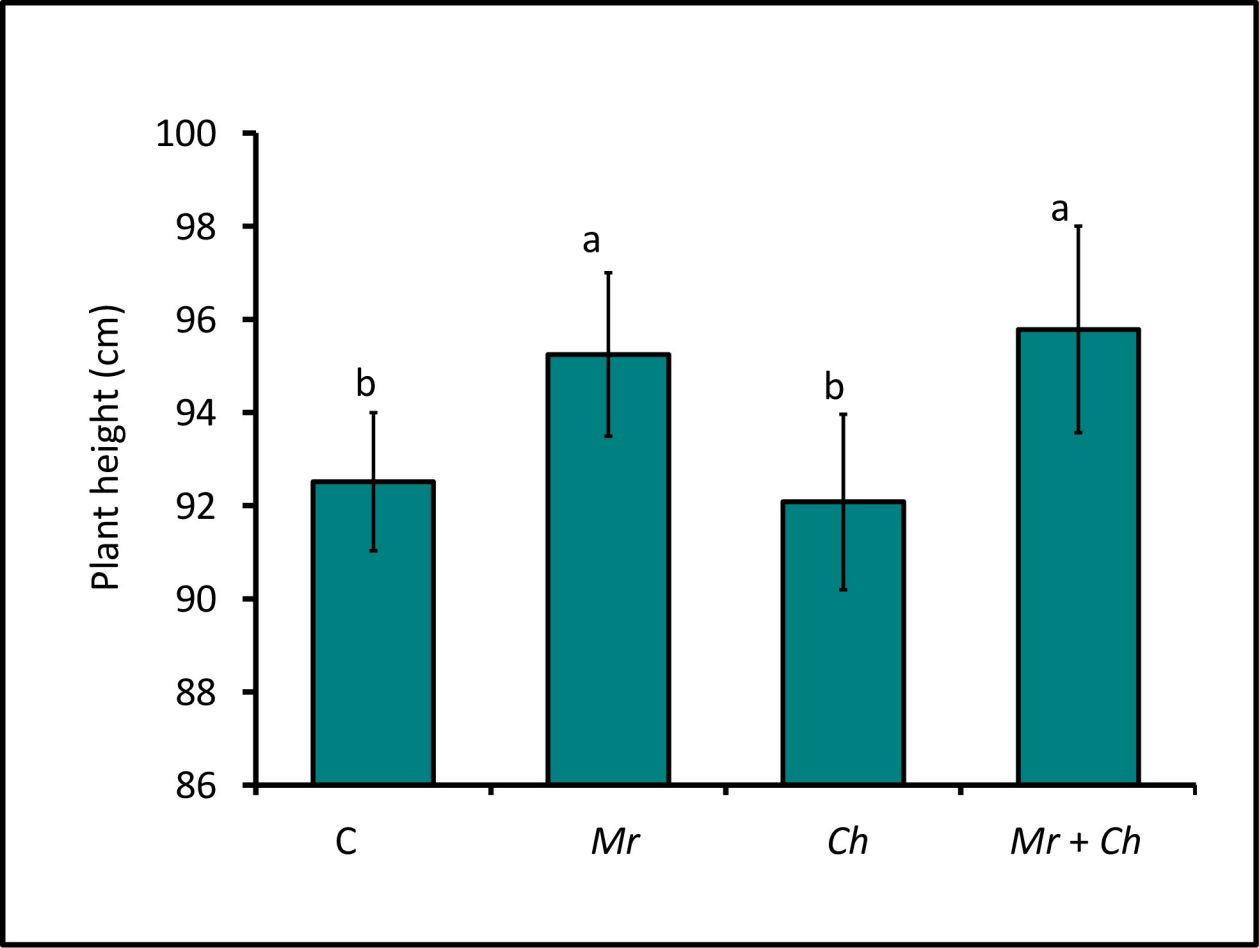
Mean height of V4 maize. End-of-experiment height of maize in *M*. *robertsii* (Mr), *C*. *heterostrophus* (Ch), *M*. *robertsii* + *C*. *heterostrophus* (Mr + Ch) treatments and untreated control (C) (*F*_3,129_ = 3.05; *P* = 0.03). Values are untransformed means ± standard error of the mean (SEM); different letters indicate significant differences at α = 0.05.

Height of ~V4-V5 maize in the *M*. *robertsii* + *C*. *heterostrophus* (95.79 ± 2.22 cm) and *M*. *robertsii-*only (95.25 ± 1.76 cm) treatments was greater than the height of plants in the untreated control (92.51 ± 1.48 cm) and *C*. *heterostrophus-*only (92.08 ± 1.89 cm) treatment. The height of maize plants in the *C*. *heterostrophus* treatment was not different than the untreated control (Fig 1).

**Fig 2:**
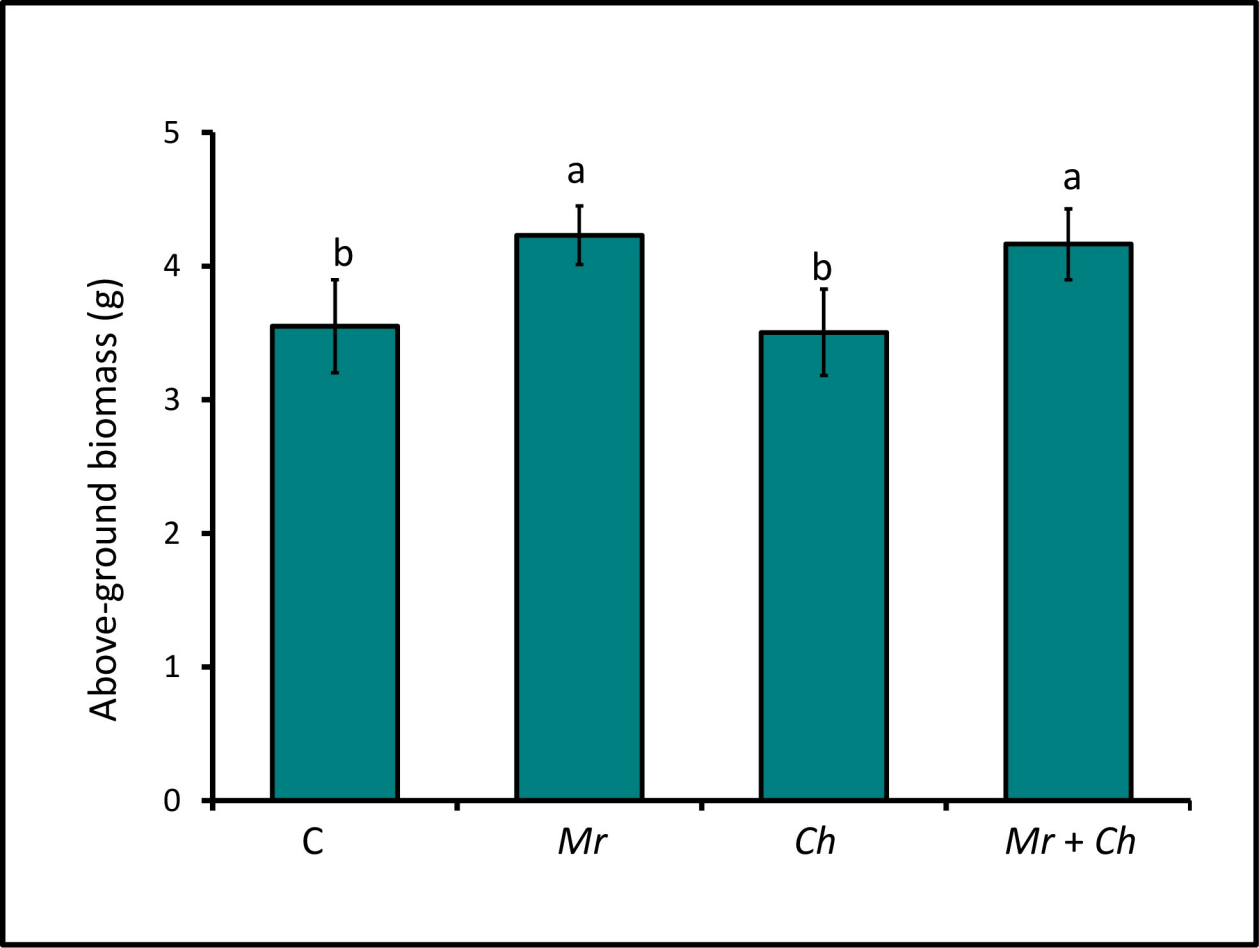
Above-ground biomass of V4 maize. End-of-experiment mean aboveground biomass of maize in the *M*. *robertsii* (Mr), *C*. *heterostrophus* (Ch), *M*. *robertsii* + *C*. *heterostrophus* (Mr + Ch) treatments and untreated control (C) (F_3,129_ = 6.05; P = 0.0007). Values are untransformed means ± SEM; different letters indicate significant differences at α = 0.05.

The above-ground biomass of ~V4-V5 maize plants in the *M*. *robertsii* + *C*. *heterostrophus* (4.16 ± 0.27 g) and *M*. *robertsii*-only (4.23 ± 0.22 g) treatments was greater than the biomass of plants in the untreated control plants (3.55 ± 0.35 g) and the *C*. *heterostrophus*-only treatments (3.5 ± 0.32 g). Biomass of plants in the *C*. *heterostrophus*-only treatment was not different from control plants. Biomass of plants in the *M*. *robertsii* + *C*. *heterostrophus* and *M*. *robertsii-*only treatments were not different (Fig 2).

### SCLB

**Fig 3:**
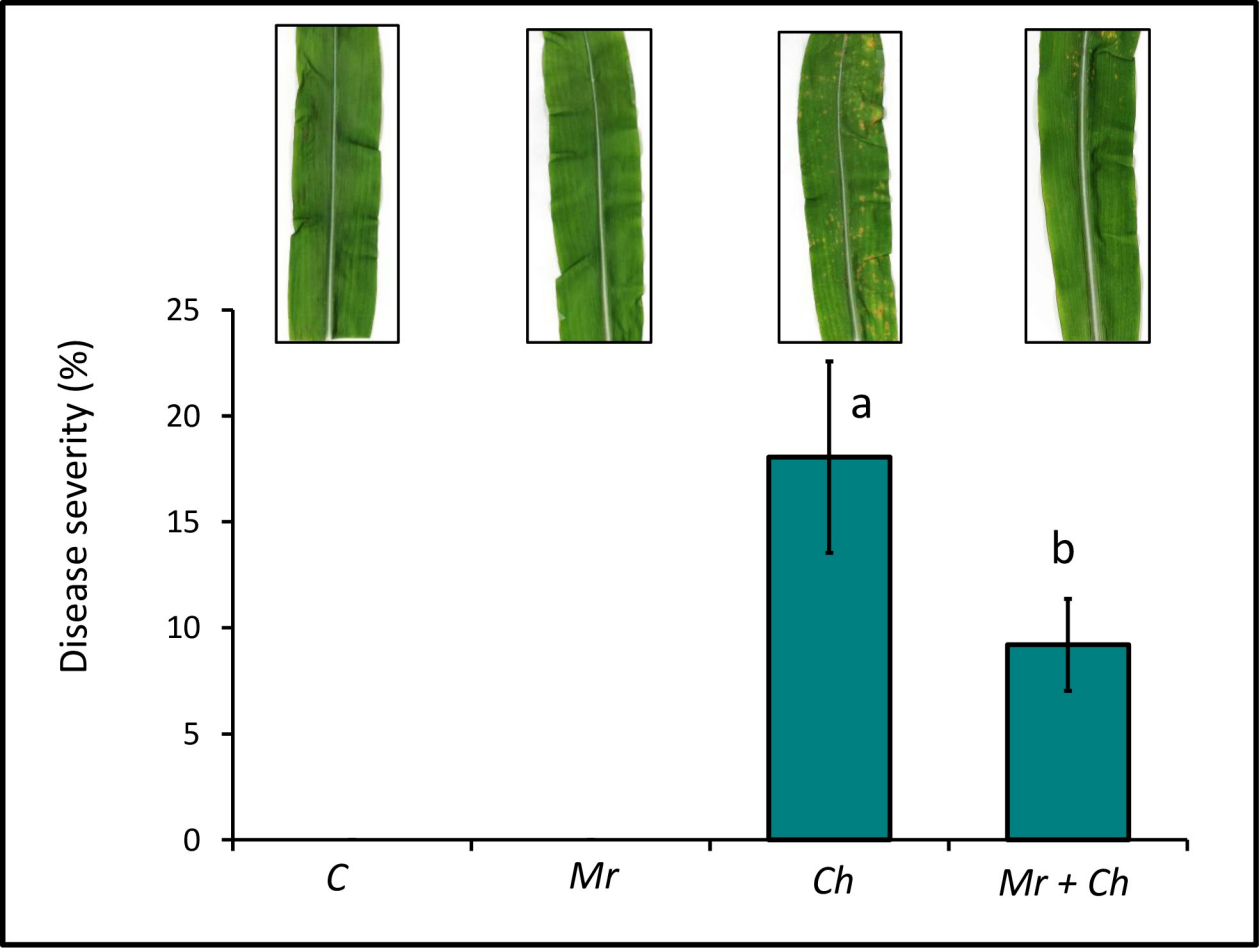
Area of diseased leaf tissue in V4 maize. End-of-experiment mean percentage of diseased maize leaf area in the *M*. *robertsii* (Mr), *C*. *heterostrophus* (Ch), *M*. *robertsii* + *C*. *heterostrophus* (Mr + Ch) treatments and untreated control (C) (*F*_3,65_ = 118; *P* <0.0001). Values are untransformed means ± SEM; different letters indicate significant differences at α = 0.05.

There was no SCLB caused by *C*. *heterostrophus* in untreated control or in plants in *M*. *robertsii*-only treatments. The percent diseased area of maize leaves in the *M*. *robertsii* + *C*. *heterostrophus* (9.2 ± 2.17%) treatment was lower than in the *C*. *heterostrophus-*only (18.05 ± 4.5%) treatment (Fig 3).

### Maize defense gene expression

#### Lipoxygenase pathway

**Fig 4:**
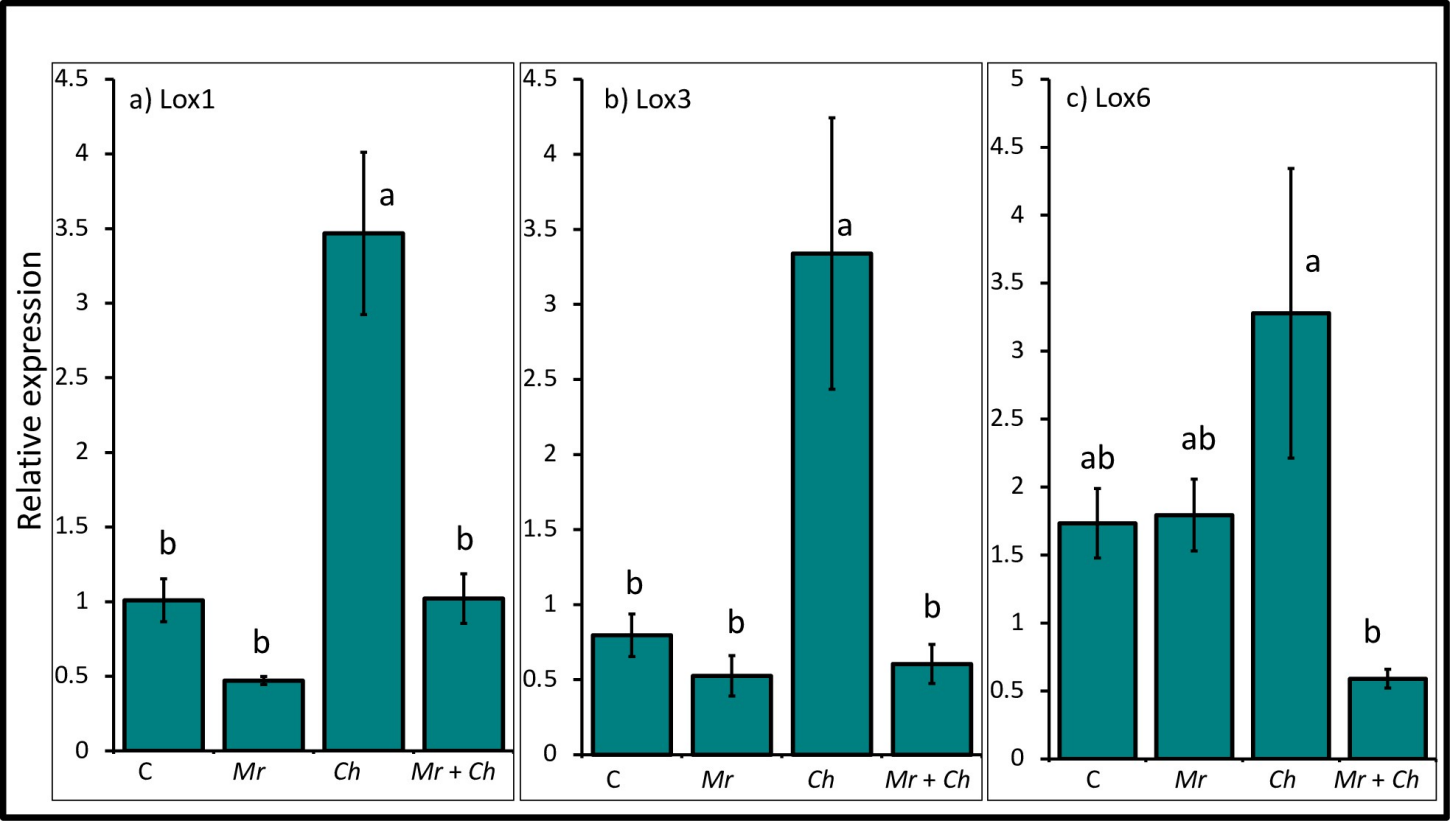
Relative expression of lipoxygenases genes. Mean relative expression of genes belonging to the JA pathway (a) *lipoxygenase 1* (*lox1*) (*F*_3,43_ = 24; *P* < 0.0001), (b) *lipoxygenase 3* (*lox3*) (*F*_3,43_ = 8.6; *P* < 0.0001), (c) *lipoxygenase 6* (*lox6*) (*F*_3,43_ = 3.8; *P* = 0.02) from 4^th^ true leaf of V4 maize in the *M*. *robertsii* (Mr), *C*. *heterostrophus* (Ch), *M*. *robertsii* + *C*. *heterostrophus* (Mr + Ch) treatments and untreated control (C). Values are untransformed means ± SEM; different letters indicate significant differences at α = 0.05.

The relative expression level of the *lipoxygenase 1 (lox1*) gene was upregulated in maize leaf tissue in plants in the *C*. *heterostrophus-*only (3.47 ± 0.54) treatment compared to the untreated control (1.01 ± 0.14), *M*. *robertsii-*only (0.47 ± 0.03) and *M*. *robertsii* + *C*. *heterostrophus* (1.2 ± 0.17) treatments. There was no difference in the expression level of *lox1* among plants in the non-inoculated control, *M*. *robertsii-*only and *M*. *robertsii* + *C*. *heterostrophus* treatments (Fig 4A).

The relative expression level of the *lipoxygenase 3 (lox3*) gene was upregulated in plants in the *C*. *heterostrophus*-only (3.34 ± 0.9) treatment compared to the non-inoculated control (0.8 ± 0.14), *M*. *robertsii-*only (0.53 ± 0.13) and *M*. *robertsii* + *C*. *heterostrophus* (0.8 ± 0.13) treatments. There was no difference in the expression level of *lox3* in the non-inoculated control, *M*. *robertsii*-only and *M*. *robertsii* + *C*. *heterostrophus* treatments (Fig 4B).

The relative expression level of *lipoxygenase 6 (lox6*) gene was upregulated in plants in the *C*. *heterostrophus*-only (3.28 ± 1.07) treatment compared to plants in the *M*. *robertsii* + *C*. *heterostrophus* (1.12 ± 1.07) treatment. There was no difference in the expression level of *lox6* among the untreated control (1.73 ± 0.26), *M*. *robertsii-*only (1.79 ± 0.26) and *M*. *robertsii* + *C*. *heterostrophus* treatments (Fig 4C).

#### Pathogenesis-related chitinases

**Fig 5:**
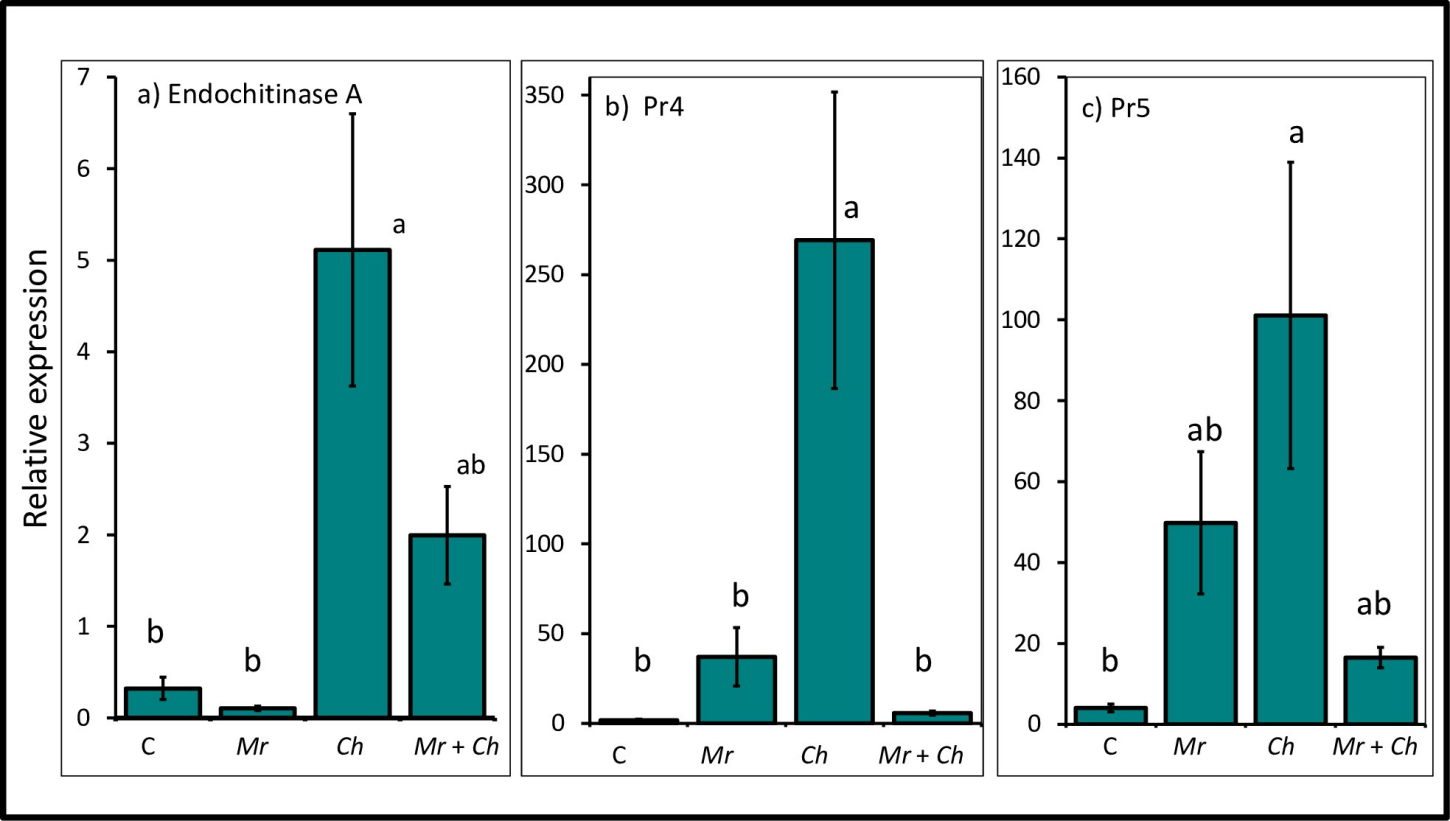
Relative expression of pathogenesis-related defense genes. Mean relative expression of (a) *endochitinase A* (*F*_3,43_ = 10.21; *P* < 0.005; (b) *pathogenesis-related gene 4* (*pr4)* (*F*_3,43_ = 11.79; *P* < 0.0001) and (c) *pathogenesis-related gene 5* (*pr5)* (*F*_3,43_ = 4.88; *P* = 0.005) from 4^th^ true leaf of V4 maize in the *M*. *robertsii* (Mr), *C*. *heterostrophus* (Ch), *M*. *robertsii* + *C*. *heterostrophus* (Mr + Ch) treatments and untreated control (C). Values are untransformed means ± SEM; different letters indicate significant differences at α = 0.05.

The expression level of *endochitinase A* was upregulated in the plants the *C*. *heterostrophus-*only (5.11 ± 1.49) treatment compared to the non-inoculated control (0.32 ± 0.12) and *M*. *robertsii*-only (0.11 ± 0.02) treatment. There was no difference in the expression level of *endochitinase A* among the non-inoculated control, *M*. *robertsii-*only and *M*. *robertsii* + *C*. *heterostrophus* (2.50 ± 0.53) treatments. There was no difference in the expression level of *endochitinase A* between the *M*. *robertsii* + *C*. *heterostrophus* and *C*. *heterostrophus-*only treatments (Fig 5A).

The relative expression level of the *pathogenesis-related gene 4 (pr4)* was upregulated in the plants in the *C*. *heterostrophus*-only (269.13 ± 82.6) treatment compared to the non-inoculated control (1.79 ± 0.64), *M*. *robertsii-*only (37.13 ± 16.22) and *M*. *robertsii* + *C*. *heterostrophus* (5.79 ± 1.14) treatments. There was no difference in the expression level of *pr4* among the non-inoculated, and the *M*. *robertsii-*only and *M*. *robertsii* + *C*. *heterostrophus* treatments (Fig 5B).

#### Pathogenesis-related proteins

The relative expression level of *pathogenesis-related gene 5 (pr5)*, a marker of the SA response pathway, was upregulated in plants in the *C*. *heterostrophus*-only (101.09 ± 37.86) treatment compared to the non-inoculated control (4.04 ± 0.97). There was no difference in the expression of *pr5* among the non-inoculated control, *M*. *robertsii*-only (49.82 ± 17.6) and *M*. *robertsii* + *C*. *heterostrophus* (16.5 ± 2.53) treatments. There was no difference in the expression level of *pr5* among plants in the *M*. *robertsii* + *C*. *heterostrophus*, *M*. *robertsii-*only and *C*. *heterostrophus*-only treatments (Fig 5C).

### Phytohormone content of maize leaf tissue

We measured the content of several growth- and defense-related phytohormones in ~V4-V5 maize leaf tissue, including cis-zeatin, gibberellins, DIMBOA (2,4-dihydroxy-7-methoxy-1,4-benzoxazin-3-one), OPDA (12-oxo-phytodienoic acid), indole acetic acid, JA, SA, and ABA. Here we present results only for those that differed among treatments.

#### Cis-zeatin

**Fig 6:**
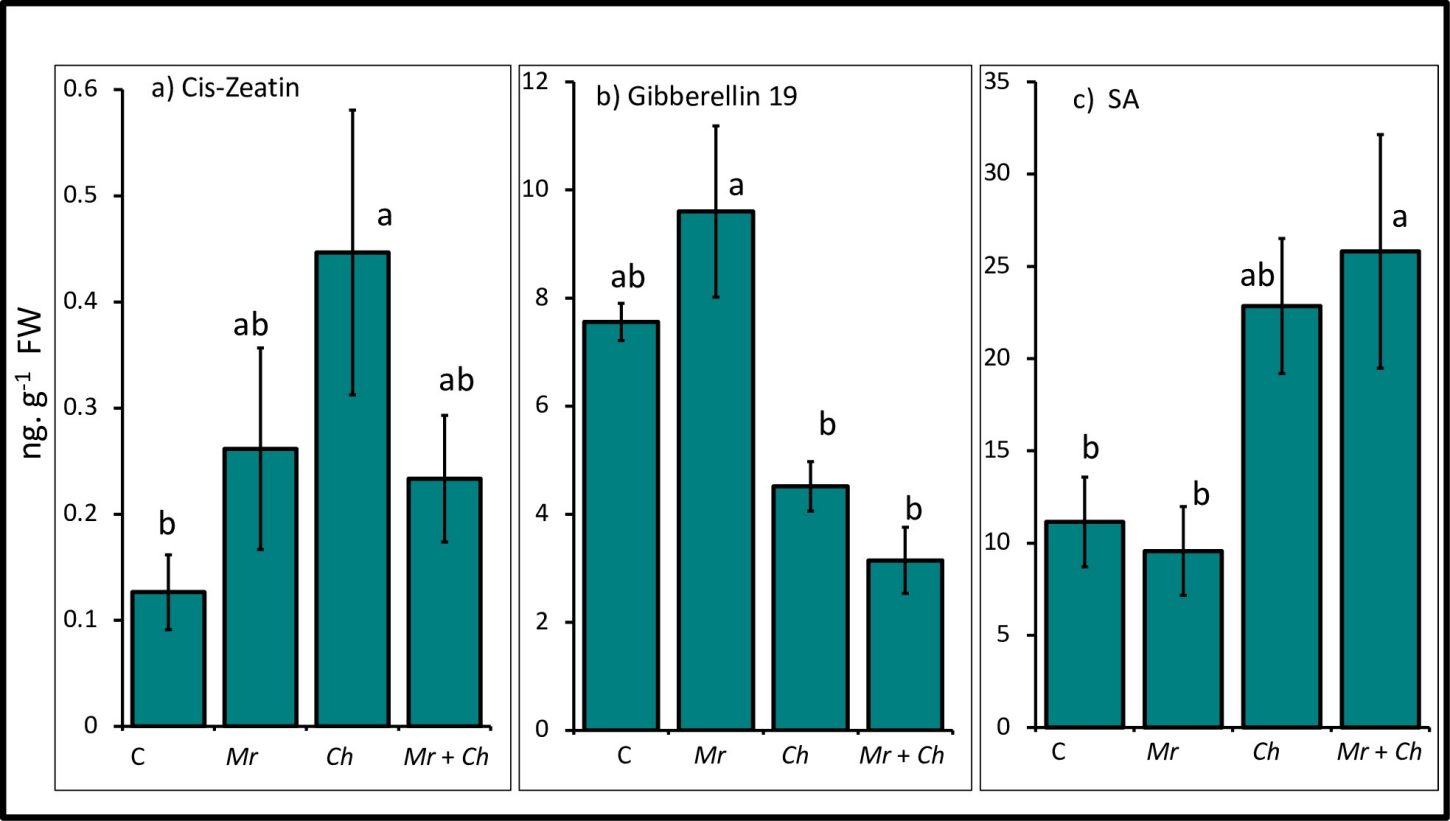
Phytohormone profiles. Mean content of the phytohormones (a) cis-zeatin (*F*_3,23_ = 3.27; *P* = 0.04), (b) gibberellin 19 (*F*_3,14_ = 14.14; *P* = 0.0002), and (c) salicylic acid (SA) (*F*_3,22_ = 5.17; *P* = 0.007) from leaf tissue of V4 maize in the *M*. *robertsii* (Mr), *C*. *heterostrophus* (Ch), *M*. *robertsii* + *C*. *heterostrophus* (Mr + Ch) treatments and untreated control (C). Values are untransformed means ± SEM; different letters indicate significant differences at α = 0.05.

Cis-zeatin content was greater in plants in the *C*. *heterostrophus*-only (0.45 ± 0.13 ng/g FW) treatment compared to the non-inoculated control (0.13 ± 0.04 ng/g FW). There was no difference in cis-zeatin among the non-inoculated control, *M*. *robertsii-*only (0.26 ± 0.1 ng/g FW) and *M*. *robertsii* + *C*. *heterostrophus* (0.23 ± 0.06 ng/g FW) treatments (Fig 6A).

#### Gibberellin 19

Gibberellin 19 content in maize leaf tissue was lower in plants in the *C*. *heterostrophus*-only (4.52 ± 0.46 ng/g FW) and *M*. *robertsii* + *C*. *heterostrophus* (3.15 ± 0.61 ng/g FW) treatments compared to *M*. *robertsii-*only (9.6 ± 1.58 ng/g FW) treatment. There was no difference in gibberellin 19 between the non-inoculated control (7.56 ± 0.04 ng/g FW) and *M*. *robertsii*-only treatment. There was no difference in gibberellin 19 between *C*. *heterostrophus-*only and *M*. *robertsii* + *C*. *heterostrophus* treatments (Fig 6B).

#### Salicylic acid (SA)

Content of SA in maize leaf tissue was greater in plants in the *M*. *robertsii* + *C*. *heterostrophus* (25.82 ± 6.33 ng/g FW) treatment compared to the non-inoculated control (11.15 ± 2.43 ng/g FW) and *M*. *robertsii*-only (9.58 ± 2.40 ng/g FW) treatment There was no difference in SA between the non-inoculated control, *M*. *robertsii*-only and *C*. *heterostrophus-*only (22.85 ± 3.66 ng/g FW) treatments. There was no difference in SA in plants in the *C*. *heterostrophus*-only and *M*. *robertsii* + *C*. *heterostrophus* treatments (Fig 6C).

## Discussion

Over the past decades, interest in exploiting beneficial soil microbes for plant growth promotion and pest and phytopathogen suppression through endophytic colonization of crops has grown rapidly [4, 75]. Soilborne endophytic insect pathogenic fungi have been the subject of extensive research since the discovery of their beneficial effects on plants when occurring as a rhizosphere inhabitant or endophyte [15]. *Metarhizium* spp. have long been studied as direct pathogens of insects and are increasingly being investigated for their indirect effects on phytopathogens through endophytic growth in host plants [19–22, 67, 76]. The mechanisms that allow plants to differentiate between colonization by mutualists and phytopathogens is still largely unknown, and our study contributes to the knowledge of differential plant immune responses to colonization by endophytic and phytopathogenic fungi.

We investigated plant growth and defense modulation in maize-*M*. *robertsii*-phytopathogen interactions. We achieved 74% colonization of maize plants grown from *M*. *robertsii-*treated seeds suggesting that seed inoculation is a reliable method for establishing *M*. *robertsii* as an endophyte of maize. We re-isolated *M*. *robertsii* in both root and leaf tissue of maize, indicating that *M*. *robertsii* established systemically. These results are consistent with studies that report systemic colonization of diverse plant species by *Metarhizium* spp. [4, 7, 15, 34, 38, 77–80].

Consistent with previous studies, maize plants grown from *M*. *robertsii-*treated seed had greater height and above-ground biomass compared to the non-inoculated control when the experiment was terminated [4, 7]. In our study, there was no difference in height and above-ground biomass of maize in response to *C*. *heterostrophus* after 96 h of inoculation. However, the absence of effects of phytopathogen infection on maize growth may have been due to the short time between inoculation with *C*. *heterostrophus* and harvest of plants for defense gene expression and phytohormone analyses.

We measured the percentage of diseased leaf area caused by *C*. *heterostrophus* in maize plants grown with and without *M*. *robertsii* treatment. There were no signs of *C*. *heterostrophus* or symptoms of SCLB disease on plants in the non-inoculated control or *M*. *robertsii-*only treatment. The percentage of area of maize leaves showing signs or symptoms of SCLB disease in the *M*. *robertsii* + *C*. *heterostrophus* treatment was lower than in the *C*. *heterostrophus-*only treatment. The lower severity of SCLB disease in plants in the *M*. *robertsii* + *C*. *heterostrophus* treatment may be due to modulation of certain plant defense pathways by endophytic *M*. *robertsii* that conferred resistance to the phytopathogen. Plants can modulate levels of phytohormones or reactive oxygen species in the presence of stress that may contribute to plant protection from phytopathogens [81]. These results are consistent with other studies that showed suppressive effects of *M*. *robertsii* against phytopathogens. For example, soil inoculation with *M*. *robertsii* resulted in reduction of disease caused by *Fusarium solani* f. sp. *phaseoli* in haricot beans [22].

We studied the expression level of genes involved in the production of lipoxygenases, enzymes involved in biosynthesis of oxylipins. Oxylipins are precursor metabolites of jasmonic acid (JA) that plays an important role in plant defense in response to herbivores, and necrotrophic and symbiotic fungi [82]. We found that the expression of lipoxygenase genes was regulated in response to colonization by *M*. *robertsii* and infection caused by *C*. *heterostrophus*. The expression levels of *lox1* and *lox3* were upregulated in plants in the *C*. *heterostrophus* treatment compared with those in the untreated control, and the *M*. *robertsii*-only and *M*. *robertsii* + *C*. *heterostrophus* treatments. Endophytic colonization by *M*. *robertsii* down-regulated the expression of *lox1* and *lox3* and their levels were equivalent to those in the control plants. The expression level of *lox6* was down-regulated in the *M*. *robertsii* + *C*. *heterostrophus* treatment compared to the *C*. *heterostrophus-*only treatment. The JA pathway is involved in the defense response against necrotrophic and symbiotic fungi in plants [83]. Plants can fine-tune their defense and growth-related pathways depending upon the nature of the challenges they face [49, 84]. In our study, the expression of lipoxygenase genes in the *M*. *robertsii* + *C*. *heterostrophus* treatment may have been down-regulated because lipoxygenases are induced in response to herbivory [82]. In the absence of herbivory, down-regulation of genes involved in herbivory-related pathways may be a strategy by plants to reduce defense and fitness costs and activate defense-related pathways against *C*. *heterostrophus* [85]. It is also possible that *M*. *robertsii*, as the first colonizer, may have down-regulated the expression of genes involved in herbivory-related pathways, and later infection by *C*. *heterostrophus* may not have had sufficient signal or power to regulate the expression of lipoxygenase genes. The plants in the *M*. *robertsii* + *C*. *heterostrophus* treatment may have used their energy to respond to signals elicited by the first colonizer, *M*. *robertsii*. Moreover, defense response of plants can involve other phytohormones such as auxins, cytokinin, brassinosteroids, and abscisic acid, through cross-communication in addition to only regulating SA, JA and ethylene pathways in modulating plant-pathogen interactions [86].

Pathogenesis-related genes are involved in plant protection against phytopathogens [87]. In addition to the evolution of other defense strategies, maize accumulates defensive proteins encoded by genes such as *endochitinase A* that suppress plant defense against herbivory but trigger defense against phytopathogens [3]. In our study, the expression levels of *endochitinase A* and *pr4* were upregulated in plants in the *C*. *heterostrophus* treatment relative to the untreated control and *M*. *robertsii*-only treatment. Higher levels of expression of *endochitinase A* and *pr4* in plants in the *C*. *heterostrophus* treatment suggest the recruitment of chitinases to degrade fungal chitin as a defense mechanism [4]. The accumulation of chitin-degrading proteins associated with the modulation of expression of these genes may confer an additional layer of defense even in the absence of herbivory. Down-regulation of *pr4* in plants in the *M*. *robertsii* + *C*. *heterostrophus* treatment may be a strategy to reduce fitness costs and mount a relatively more effective defense signal than the upregulation of *pr4* [3].

We measured the expression level of *pr5*, a marker of the SA response pathway. Expression of *pr5* was upregulated in plants in the *C*. *heterostrophus* treatment compared to the non-inoculated control. There was no difference in the expression level of *pr5* gene among the non-inoculated control, and *M*. *robertsii* and *M*. *robertsii* + *C*. *heterostrophus* treatments. The higher level of *pr5* expression in the *C*. *heterostrophus* treatment suggests that maize plants perceived and responded to infection by accumulation of *pr5* to activate the SA-dependent pathway [50]. Our study is consistent with other reports on the regulation of the SA-dependent pathways in response to infection by phytopathogens in maize [54].

Phytohormones are among the key players in modulating plant defense signaling through positive and negative interactions for efficient stress management through induced systemic resistance (ISR) or systemic acquired resistance (SAR) [47]. Furthermore, pathogens can affect defense signaling networks of plants for their own benefit through phytohormone homeostasis [88]. In our study, we evaluated growth- and defense-related phytohormone content in maize leaf tissue. There was no difference in the JA content among different treatments (data not reported). This could be due to the absence of herbivory in our experiment as the JA-dependent pathway is primarily a defense response against herbivores. Content of cis-zeatin, a cytokinin involved in plant defense against various biotic and abiotic stresses, was greater in plants in the *C*. *heterostrophus* treatment compared to the non-inoculated control, whereas there was no difference in cis-zeatin among the non-inoculated control, and the *M*. *robertsii-*only and *M*. *robertsii* + *C*. *heterostrophus*-treatments. These results suggest that *C*. *heterostrophus* may have induced the production of cis-zeatin, whereas the suppression of cis-zeatin in the *M*. *robertsii* + *C*. *heterostrophus* treatment may be a defense strategy to activate other, relatively more aggressive, signaling pathways for a better induction of defense [89]. In another study, cis-zeatin and trans-zeatin differentially suppressed the infection caused *Pseudomonas syringae* in tobacco where trans-zeatin induced a stronger immune response [90]. Cis-zeatin is considered less active compared to its trans isomer and plays a significant role in plant defense against phytopathogens [90]. Plants in the *M*. *robertsii* + *C*. *heterostrophus* treatment may not have induced the biosynthesis of cis-zeatin in response to *C*. *heterostrophus* because of the energy required to respond to the earlier establishment of *M*. *robertsii*.

Gibberellins are phytohormones involved in regulation of plant growth and development. In our study, gibberellin 19 content was lower in plants in the *C*. *heterostrophus* and *M*. *robertsii* + *C*. *heterostrophus* treatments compared with those in the *M*. *robertsii*-only treatment. There was no difference in gibberellin 19 content between the non-inoculated control and *M*. *robertsii*-only treatment or between the *C*. *heterostrophus*- and *M*. *robertsii* + *C*. *heterostrophus* treatments. The higher level of gibberellin 19 in plants in the *M*. *robertsii*-only treatment and lower level in plants in the *C*. *heterostrophus* and *M*. *robertsii* + *C*. *heterostrophus* treatments may be due to the activation of plant growth promotion pathways by *M*. *robertsii* and growth suppressive effects of *C*. *heterostrophus*, respectively. Some endophytes produce gibberellins and auxins *in planta* that promote plant growth [62, 91, 92]. Hu and Bidochka (2021) reported that combined or separate inoculation with either *M*. *robertsii* or the phytopathogenic *F*. *solani* did not induce any changes in gibberellin content in common bean leaf tissue compared with non-inoculated control plants [19].

Plants can regulate their growth and defense by the SA-dependent pathway, which is involved in defense against biotrophic phytopathogens and phloem feeding insects [50]. In our study, SA content was greater in plants in the *M*. *robertsii* + *C*. *heterostrophus* treatment compared to the non-inoculated control and *M*. *robertsii*- only treatment. There was no difference in SA content in plants among the non-inoculated control, *M*. *robertsii*-only and *C*. *heterostrophus*-only treatments. Nor was there a difference in SA content between the plants in the *C*. *heterostrophus* and *M*. *robertsii* + *C*. *heterostrophus* treatments. The higher level of SA in plants in the *M*. *robertsii* + *C*. *heterostrophus* treatment may be due to the cumulative response against *M*. *robertsii* and *C*. *heterostrophus* where plants may have perceived *M*. *robertsii* as a biotrophic invader and responded by eliciting the SA response pathway [50]. Our results are consistent with other studies that reported modulation of the level of SA in response to endophytic *M*. *anisopliae* in maize and fungal phytopathogens [19, 54].

Mounting a defense response by plants in response to stresses involves ISR and SAR, which are regulated by complex interactions of signaling molecules in which phytohormones play a central role [47] and can be induced by phytopathogens, chemical inducers, insect herbivores or specific root-colonizing microbes, such as mycorrhizae and rhizobacteria [48, 93]. ISR- and SAR- related defense involves JA- and SA-dependent pathways [93–96] wherein the regulation of phytohormone gene expression acts a defensive strategy against different stresses that allows successful establishment of symbioses [95, 97].

We found that endophytic colonization of maize by *M*. *robertsii* increased plant growth, possibly through better nutrient acquisition or assimilation mediated through the activation of plant growth-related signaling pathways. Endophytic *M*. *robertsii* reduced the severity of SCLB compared to the non-endophytic maize plants, perhaps due to disease resistance caused by accumulation of anti-fungal compounds mediated through the establishment of *M*. *robertsii* as an endophyte. We observed that endophytic colonization by *M*. *robertsii* down-regulated the expression of pathogenesis-related genes. It is possible that endophytic colonization may have resulted in the down-regulation of certain pathogenesis-related genes but may have induced the upregulation of other defense-related pathways through hormonal cross talk for a better defense against SCLB. We found that endophytic *M*. *robertsii* down-regulated the expression of lipoxygenases. Because lipoxygenases are usually involved in plant defense against herbivory, *M*. *robertsii* may have induced the down-regulation of lipoxygenases for balancing the trade-off between plant growth and defense in the absence of herbivory. In our study, we also found that the level of SA was greater in plants in the *M*. *robertsii* + *C*. *heterostrophus* treatment compared to the non-inoculated control. Although endophytic *M*. *robertsii* down-regulated the expression of lipoxygenases and pathogenesis-related genes in maize, SA content was greater in endophytically colonized plants. This may be due to the positive and negative cross talk of other defense signaling pathways that we may not have addressed in this study. Our study suggests that endophytic colonization by *M*. *robertsii* may have increased growth of maize plants by induction of gibberellins. However, infection by *C*. *heterostrophus* may have had an adverse effect on growth if we had extended the time between inoculation of plants with *C*. *heterostrophus* and termination of the experiment. Our study suggests a potential mechanism of suppression of SCLB by endophytic *M*. *robertsii* in maize is through induction of greater SA content compared to non-inoculated control plants. It highlights the direct or indirect mechanistic effects of endophytic *M*. *robertsii* on the modulation of pathogenesis- and growth-related phytohormones and expression of genes in plants subsequently infected with the phytopathogen, *C*. *heterostrophus*.

Several challenges remain to be explored before endophytic insect pathogens, such as *Metarhizium* spp., can be predictably exploited in the field for pest management. For example, information critical for the deployment of this approach, such as the variability of ecological competency among species and isolates of *Metarhizium* in agricultural soils, the prevalence and persistence of natural and managed endophytic colonization, and mechanisms of action of plant-growth promoting and disease suppressive effects of endophytic *Metarhizium* spp. remain to be better understood.

## Conclusion

To conclude, through seed inoculation we established endophytic colonization of maize root and foliar tissue by *M*. *robertsii* that resulted in plant growth promotion, SCLB disease suppression and changes in phytohormone content and defense gene expression.

## Supporting information

S1 Table Gene sequences of the primers used for qRT-PCR for maize. (DOCX)
