# Supplementary material for: Endophytic Metarhizium robertsii suppresses the phytopathogen, Cochliobolus heterostrophus and modulates maize defenses
Source: PLoS One. 2022 Sep 22;17(9):e0272944. doi: 10.1371/journal.pone.0272944 (PMC9499252; doi:10.1371/journal.pone.0272944)
Supplement: S1 Table — (DOCX) [file pone.0272944.s001.docx]

**Table S1:** Gene sequences of the primers used for qRT-PCR for maize.

| **Gene** | **Forward primer** | **Reverse primer** | **Accession number** | **Reference** |
| --- | --- | --- | --- | --- |
| Actin | GGAGCTCGAGAATGCCAAGAGCAG | GACCTCAGGGCATCTGAACCTCTC | U60511.1 | [1] |
| Pathogenesis-related gene 5 (*Pr5*) | tgcatgcatgggctagtgat | cgcacacaaatccagctacg | U82201 | [2] |
| Pathogenesis-related gene 4 (*Pr4)* | GCCAACCACGAGACCATAAAG | CACGGCCACTGCGTGTT | NP_001150754.1 | [3] |
| Endochitinase A | CAAGACGGCGCTCTGGTT | AAGCCCTGCGGCATCA | NP_001158904.1 | [3] |
| Lipoxygenase 1 *(Lox1)* | CGTTCCGTGAAGTGTGGTTCT | CTGTAAGGAGTACTTGGCATATTTGC | AF271894 | [4] |
| Lipoxygenase 3 (*Lox3*) | TCACGAGCCAGATCCAGACCA | ATTCGATTCACCAGCCCACACG | AF149803 | [4] |
| Lipoxygenase 6 (*Lox6*) | ACAGCCCTGACTGGTGCTC | TTCACGTTTATGTGGTGGAGA |  | [5] |

1. Moniz de Sa M, Drouin G. Phylogeny and substitution rates of angiosperm actin genes. Mol Biol Evol. 1996;13: 1198–1212.

2. Morris SW, Vernooij B, Titatarn S, Starrett M, Thomas S, Wiltse CC, et al. Induced resistance responses in maize. Mol Plant-Microbe Interact. 1998;11: 643–658.

3. Schnable PS, Ware D, Fulton RS, Stein JC, Wei F, Pasternak S, et al. The B73 maize genome: complexity, diversity, and dynamics. Science (80- ). 2009;326: 1112–1115.

4. Shivaji R, Camas A, Ankala A, Engelberth J, Tumlinson JH, Williams WP, et al. Plants on constant alert: elevated levels of jasmonic acid and jasmonate-induced transcripts in caterpillar-resistant maize. J Chem Ecol. 2010;36: 179–191.

5. Christensen SA, Huffaker A, Kaplan F, Sims J, Ziemann S, Doehlemann G, et al. Maize death acids, 9-lipoxygenase–derived cyclopente (a) nones, display activity as cytotoxic phytoalexins and transcriptional mediators. Proc Natl Acad Sci. 2015;112: 11407–11412.
